# Oropharynx and hyoid bone changes in female extraction patients with distinct sagittal and vertical skeletal patterns: a retrospective study

**DOI:** 10.1186/s13005-022-00334-1

**Published:** 2022-09-05

**Authors:** Runzhi Guo, Shuo Wang, Liwen Zhang, Linwei Li, Qianyao Yu, Yiping Huang, Weiran Li

**Affiliations:** 1 Department of Orthodontics, Peking University School and Hospital of Stomatology, 22 Zhongguancun Avenue South, Haidian District, Beijing, 100081 P.R. China; 2 Department of Dental Medical Center, China-Japan Friendship Hospital, Beijing, China

**Keywords:** Extraction, Oropharynx, Hyoid bone, Cone beam computed tomography

## Abstract

**Background:**

Previous studies have reported inconsistent effects of premolar extraction on the oropharynx and hyoid bones. Currently, no strong evidence is available regarding the effect of extraction on upper airway size. Hence, the aim of this study was to analyse the effects of first premolar extraction on the oropharynx and hyoid bone positions in female adult patients, and further explored differences in oropharynx and hyoid bone changes among skeletal patterns.

**Methods:**

The study population included 40 female adult patients who did not undergo extraction and 120 female adult patients who underwent extraction of four premolars; the including patients had four distinct sagittal and vertical skeletal patterns. Cone-beam computed tomography was performed before (T0) and after (T1) orthodontic treatment. Eight oropharynx variables and five hyoid bone variables were measured using Dolphin 3D Imaging software. Paired and independent t-tests were used to analyse measurements between timepoints and groups, respectively.

**Results:**

The oropharynx volume increased significantly in the extraction group; changes did not differ significantly between extraction and non-extraction groups. Oropharynx variables did not differ significantly at T0 among the four skeletal pattern groups. After orthodontic extraction treatment, the oropharynx volume increased significantly in the class I-norm and class I-hyper subgroups, but not in the class II-norm and class II-hyper subgroups. Significant increases were observed in the oropharynx volume and most constricted axial area from T0 to T1 in the moderate retraction group, but not in the maximum retraction group. Extraction patients exhibited significant posterior movement of the hyoid, particularly among maximum retraction patients.

**Conclusions:**

In female adult patients, first premolar extraction tends to increase the oropharynx size and cause posterior movement of the hyoid bone, particularly in skeletal class I patients. For skeletal class II and hyperdivergent patients with a narrow oropharynx, first premolar extraction does not negatively influence oropharynx size or hyoid bone position. The differences of oropharyngeal changes between moderate retraction patients and maximum retraction patients were not significant.

**Supplementary Information:**

The online version contains supplementary material available at 10.1186/s13005-022-00334-1.

## Background

The goals of orthodontic treatment are aesthetics, stability, and function. Dental extractions are commonly used to provide spacing that resolves dental crowding; extractions can also retract proclined anterior teeth. Dental and skeletal changes after extraction treatment have consistently achieved stable and acceptable aesthetic outcomes [1, 2]. Recently, more attention has focused on upper airway changes in extraction patients. The upper airway consists of the nasopharynx, oropharynx, and hypopharynx. Among these, the oropharynx is mainly surrounded by soft tissues, such as the soft palate and tongue; it is directly connected to the oral cavity. Hence, oropharynx dimensions are easily influenced by extraction treatment. The hyoid bone is a U-shaped bone connected with the pharynx, cranial base, and mandibular symphysis via muscles and ligaments. A close relationship has been identified between the upper airway and hyoid bone; the hyoid bone can adjust its position to maintain upper airway stability [3, 4].

Although the effects of dental extraction on airway and hyoid bone have been investigated in previous studies, the results have been inconsistent. Some authors have reported that extraction-induced anterior tooth retraction can decrease the oral cavity volume and change the positions of the tongue and hyoid bone, leading to a narrow oropharynx [5–7]. In contrast, Maaitah et al. and Stefanoivc et al. found no significant changes in oropharynx size or hyoid bone position after orthodontic treatment involving the extraction of four premolars [8, 9]. Zhang et al. found that self-regulation of the upper airway could maintain airway size during extraction treatment [10]. At present, no strong evidence is available regarding the effects of extraction on upper airway size. The inconsistent results to date could be attributed to differences in patient age, sex, skeletal pattern, and extraction indication. Some previous studies have reported on the relationships between the oropharynx and craniofacial skeletal morphology [11–13]. The oropharynx could be affected by craniofacial skeletal morphology. Some skeletal patterns (e.g. skeletal class II, hyperdivergent patterns) are presumably associated with a narrow oropharynx. To our knowledge, the effects of first premolar extraction on oropharynx size and hyoid bone position have not been investigated in patients with various skeletal patterns.

Most previous studies regarding the effects of extraction on the upper airway have been performed using lateral cephalography [6–8, 14, 15]. Sears et al. found weak correlations between oropharynx linear and volume measurements, on the basis of lateral cephalogram and cone-beam computed tomography (CBCT) findings [16]. Compared with the anteroposterior airway information provided by lateral cephalography, CBCT enables three-dimensional imaging of the upper airway, along with analyses of its morphology and volume. To eliminate the confounding effects of growth, sex, and skeletal differences, this retrospective study used CBCT to analyse the effects of first premolar extraction on oropharynx size and hyoid bone position in female adults; it also explored differences in oropharynx size and hyoid bone changes among skeletal patterns.

## Materials and methods

### Participants

The protocol for this retrospective study was approved by the Peking University School and Hospital of Stomatology ethics committee (PKUSSIRB-2013029). The study population included patients treated at the Department of Orthodontics, Peking University School and Hospital of Stomatology between June 2014 and June 2021. Inclusion criteria were as follows: female sex; age 20–50 years; skeletal class I and class II occlusion with average and high mandibular angles (ANB > 0°, SN-MP > 28°); mild to moderate crowding; fixed appliance treatment; body mass index < 27 kg/m^2^ at pre-treatment; no breathing disorder; no cleft lip and/or palate; and no craniofacial syndromes. All patients underwent extraction of the four first premolars to resolve crowding and retract the anterior teeth (the maxillary anterior teeth retraction ≥3 mm). The patients with class II division 2 were excluded due to the limited anterior teeth retraction. Non-extraction (control) patients were selected by age and skeletal type matching. To minimise the effects of head position on the oropharynx, patients with non-natural head positions during CBCT were excluded from the study; patients with poor CBCT scan quality were also excluded. Finally, 120 female extraction patients and 40 female non-extraction patients were enrolled in the study. All patients provided written informed consent to participate.

### Groups

The 120 female extraction patients were further divided into four subgroups based on their ANB and SN-MP characteristics:

- Class I-norm: 30 patients with skeletal class I occlusion and normodivergence (0° < ANB < 5°, 28° < SN-MP < 37°)
- Class I-hyper: 30 patients with skeletal class I occlusion and hyperdivergence (0° < ANB < 5°, SN-MP ≥ 37°)
- Class II-norm: 30 patients with skeletal class II occlusion and normodivergence (ANB ≥ 5°, 28° < SN-MP < 37°)
- Class II-hyper: 30 patients with skeletal class II occlusion and hyperdivergence (ANB ≥ 5°, SN-MP ≥ 37°)

The matched non-extraction group consisted of 10 Class I-norm patients, 10 Class I-hyper patients, 10 Class II-norm patients and 10 Class II-hyper patients.

### CBCT

CBCT scans were obtained before (T0) and after (T1) orthodontic treatment using a NewTom Scanner (Marburg, Germany) with the following parameters: axial slice thickness, 0.3 mm; field of view, 13 cm × 17 cm; and scan time, 15 s. During scanning sessions, patients sat upright; they were instructed to bite in centric occlusion and breathe normally without swallowing. Each patient’s natural head position was adjusted by an experienced radiologist to ensure that the orbital-auricular plane was parallel to the floor. All CBCT data were exported into DICOM format and analysed using Dolphin 3D Imaging software (version 11.8, Dolphin Imaging and Management Solutions, Chatsworth, Calif). CBCT images were oriented as follows: the horizontal plane passed through the left/right orbitales and the right porion; the sagittal plane passed through the nasion and anterior nasal spine. The coronal plane was perpendicular to both horizontal and sagittal planes. Lateral cephalographs were reconstructed from CBCT scans using Dolphin 3D Imaging software for hyoid measurement and cephalometric analysis.

### Measurements

#### Oropharynx measurement

**Fig. 1:**
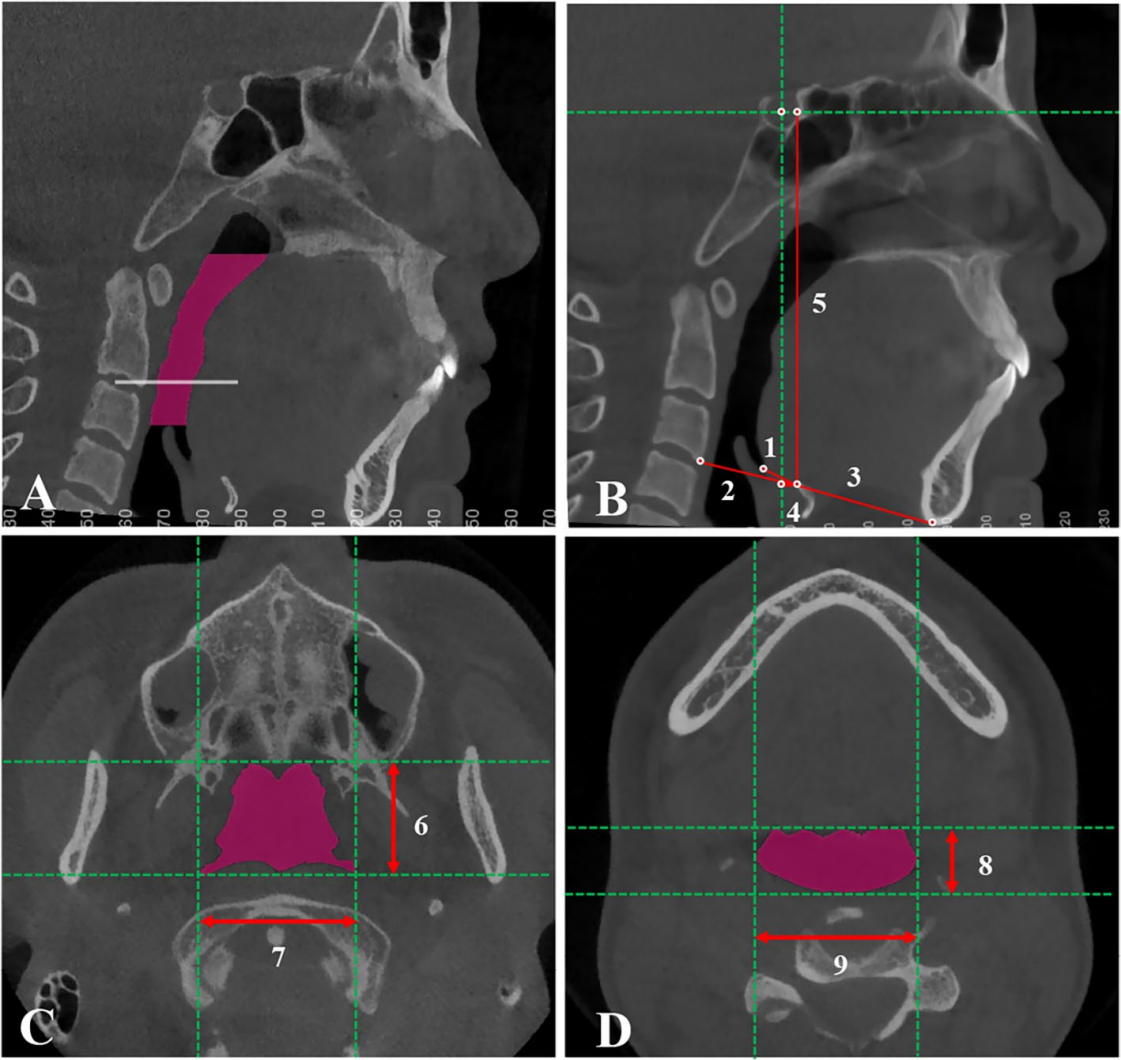
Measurements of oropharynx size and hyoid bone position. **A** Volume and most constricted axial (MCA) area of the oropharynx. **B** Five hyoid bone measurements: H-Eb (1), H-C3 (2), H-Me (3), H-X (4), and H-Y (5). **C** Anterior-posterior (6) and lateral (7) diameters of the oropharynx at the level of the PNS plane. **D** Anterior-posterior (8) and lateral (9) diameters of the oropharynx at the level of the epiglottis tip

The oropharynx in CBCT was defined as the region from the plane passing the posterior nasal spine to the plane passing the tip of the epiglottis [17]. The volume and the most constricted axial (MCA) area of the oropharynx were calculated (Fig. 1A). Airway segmentation threshold values ranging from 50 to 70 were adjusted to discriminate soft tissue from the airway. As shown in Fig. 1C and D, the anterior-posterior (AP) diameter, lateral diameter, and ratio of AP/lateral diameter were also measured at the level of the PNS plane and the tip of the epiglottis (E) to analyse the morphology of the oropharyngeal airway.

#### Hyoid measurement

As shown in Fig. 1B, five linear parameters were selected to measure the hyoid bone position: H-Eb (highest point of hyoid bone to the epiglottis base); H-C3 (highest point of hyoid bone to the most anterior and inferior point of the third cervical vertebra); H-Me (highest point of hyoid bone to the menton); H-X (perpendicular distance from the highest point of hyoid bone to the vertical line passing through the sella); and H-Y (perpendicular distance from the highest point of hyoid bone to the horizontal line passing through the sella).

#### Cephalometric analysis

SNA, SNB, ANB, and SN-MP angles were selected as skeletal measurements. U1-SN, L1-MP, L1-X (perpendicular distance from the mandibular central incisor incisal edge to the vertical line passing through the sella), and U1-X (perpendicular distance from the maxillary central incisor incisal edge to the vertical line passing through the sella) were selected as dental measurements. Patients were grouped based on these skeletal and dental measurements, as shown in Table 1.

**Table 1:** The baseline demographic information and cephalometric analysis of included patients

	Extraction group (***n*** = 120)				Non-extraction group (***n*** = 40) Mean (SD)
	Class I-norm (***n*** = 30) Mean (SD)	Class I-hyper (***n*** = 30) Mean (SD)	Class II-norm (***n*** = 30) Mean (SD)	Class II-hyper (***n*** = 30) Mean (SD)	
**Demographic**					
Age, years	24.8 (3.6)	24.3 (4.1)	26.1 (2.9)	25.1 (3.2)	26.4 (4.9)
BMI, kg/m^2^	21.2 (1.2)	22.4 (2.1)	20.8 (1.8)	21.1 (1.8)	22.1 (2.4)
Treatment duration, years	2.6 (0.6)	2.5 (0.4)	2.7 (0.3)	2.7 (0.4)	2.0 (0.3)
**Cephalometric analysis**					
SNA	82.3 (1.6)	81.5 (1.3)	81.1 (2.2)	83.2 (2.1)	82.1 (3.2)
SNB	79.4 (2.4)	78.1 (2.3)	74.8 (2.7)	75.2 (3.2)	76.9 (2.2)
ANB	2.9 (1.2)	3.4 (1.1)	5.9 (1.8)	6.7 (1.9)	5.2 (1.7)
SN-MP	32.3 (3.0)	43.2 (3.1)	34.1 (2.1)	41.9 (2.4)	37.2 (3.8)
U1-SN	115.7 (4.2)	112.2 (3.9)	106.2 (5.2)	104.2 (4.4)	101.2 (6.3)
L1-MP	97.6 (5.6)	98.3 (6.2)	99.4 (4.2)	102.1 (6.8)	94.2 (7.9)
U1-X, mm	63.9 (3.5)	64.7 (4.2)	64.1 (3.9)	65.2 (2.9)	62.8 (3.8)
L1-X, mm	61.4 (4.3)	61.7 (5.1)	60.2 (6.0)	61.3 (4.7)	61.2 (2.7)

### Statistical analysis

All measurements were performed by an experienced orthodontist. To assess reliability, 32 of the included patients were randomly selected; all measurements of these patients were repeated after an interval of 2 weeks. The intra-class correlation coefficients of all measurements were 0.872–0.954, indicating good reliability. The method error was measured using the Dahlberg formula. The method error ranged from 0.11 to 0.34 mm for linear measurements, from 0.17° to 0.48° for angular measurements, from 6.2 to 22.1 mm^2^ for area measurements, and from 77.2 to 212.4 mm^3^ for volume measurements. The normalities of measurement distributions were assessed using the Shapiro–Wilk test. For normally distributed measurements, paired and independent t-tests were used to analyse measurements between timepoint and groups, respectively. When measurement distributions were not normally distributed, the Wilcoxon signed-rank test was used. The statistical significance was set at *P* < 0.05. All statistical analyses were performed using SPSS software (version 26; SPSS, Chicago, IL, USA).

## Results

### Comparisons of oropharynx and hyoid bone changes between extraction and non-extraction groups

Table 2 lists changes in oropharynx and hyoid bone positions in the extraction and non-extraction groups. In the extraction group, the oropharynx volume increased significantly from T0 to T1; the MCA of oropharynx slightly increased, but without significance. The PNS-AP, PNS-lateral, and E-lateral also increased significantly in the extraction group. In the non-extraction group, the volume and MCA of oropharynx were stable after treatment. Oropharynx changes did not differ significantly between extraction and non-extraction groups. With regard to the hyoid bone, the H-X distance decreased and H-Me distance increased significantly in the extraction group, indicating posterior movement of the hyoid bone. In the non-extraction group, no significant change was found for all hyoid bone variables. Changes in H-Me and H-X distances differed significantly between groups. Considering the potential influence of skeletal pattern, we further compared the oropharynx and hyoid bone changes between extraction and non-extraction patients with four skeletal patterns (Supplementary Tables 1, 2, 3 and 4). In all subgroups, the oropharynx changes were not significant between extraction and non-extraction patients. In class I-hyper subgroup, the H-X distance decreased in extraction patients and increased in non-extraction patients, which the difference was significant.

**Table 2:** Comparison of oropharynx and hyoid bone position between extraction and non-extraction groups

Variable	Extraction group (***n*** = 120)			Non-extraction group (***n*** = 40)			Extraction group vs Non-extraction group
	T0 Mean (SD)	T1 Mean (SD)	***P***	T0 Mean (SD)	T1 Mean (SD)	***P***	***P***
**Oropharynx**							
Vol, mm^3^	16,123.7 (5510.6)	17,782.1 (6366.4)	0.000*	16,445.1 (5616.7)	16,621.2 (5626.2)	0.808	0.163
MCA, mm^2^	243.3 (112.6)	266.7 (124.8)	0.060	236.4 (104.9)	233.9 (99.2)	0.757	0.246
PNS-AP	27.7 (3.7)	28.2 (2.7)	0.002*	28.2 (4.2)	28.8 (3.6)	0.182	0.062
PNS-lateral	37.8 (5.7)	40.0 (6.8)	0.000*	38.9 (5.9)	40.0 (5.7)	0.08	0.613
PNS-AP/ lateral	0.72 (0.10)	0.71 (0.10)	0.551	0.73 (0.07)	0.73 (0.08)	0.645	0.912
E-AP	13.1 (3.2)	13.2 (3.8)	0.446	12.4 (3.6)	12.6 (3.7)	0.624	0.325
E- lateral	30.4 (2.9)	31.3 (3.3)	0.003*	31.1 (3.5)	30.9 (3.9)	0.668	0.649
E-AP/lateral	0.41 (0.10)	0.42 (0.10)	0.701	0.40 (0.10)	0.41 (0.11)	0.392	0.575
**Hyoid**							
H-Eb	8.4 (1.7)	8.5 (2.1)	0.93	9.1 (2.6)	8.7 (2.6)	0.283	0.283
H-Me	44.2 (4.6)	45.7 (5.7)	0.002*	45.9 (4.8)	45.2 (4.9)	0.384	0.014*
H-C3	27.6 (3.2)	27.7 (3.2)	0.151	27.9 (3.3)	27.4 (3.7)	0.252	0.189
H-X	8.8 (7.1)	7.1 (7.0)	0.005*	7.8 (7.2)	8.9 (7.1)	0.257	0.014*
H-Y	93.4 (5.6)	93.8 (5.3)	0.123	95.7 (6.7)	93.8 (12.7)	0.586	0.294

^*^P < 0.05

### Comparisons of oropharynx and hyoid bone changes among extraction patients with distinct skeletal patterns

Table 3 compares oropharynx and hyoid bone at T0 among the four skeletal pattern subgroups. None of the oropharynx variables differed significantly among the four subgroups at T0. The oropharynx volume tended to be smaller in class II-hyper patients than in the other three skeletal pattern subgroups, and the MCA tended to be smaller in the Hyperdivergent group (class I-hyper and class II-hyper) than in the Normodivergent group (class I-norm and class II-norm), but these differences were not statistically significant. With regard to the hyoid bone, the skeletal patterns differed significantly according to H-X distance. The H-X was significantly smaller in class II-hyper patients than in the other three skeletal pattern subgroups, indicating a posterior hyoid bone position.

**Table 3:** Comparison of pretreatment oropharynx and hyoid bone position in extraction patients with different skeletal patterns

Variable	Class I-norm	Class I-hyper	Class II-norm	Class II-hyper		Class I-norm vs Class I-hyper	Class I-norm vs Class II-norm	Class I-norm vs Class II-hyper	Class I-hyper vs Class II-norm	Class I-hyper vs Class II-hyper	Class II-norm vs Class II-hyper
	T0 Mean (SD)	T0 Mean (SD)	T0 Mean (SD)	T0 Mean (SD)	Overall *P*^*a*^	***p***	***p***	***p***	***p***	***p***	***p***
**Oropharynx**											
Vol, mm^3^	16,393.0 (5127.0)	16,270.2 (5429.6)	16,708.3 (6318.0)	15,123.3 (5238.0)	0.844	0.446	0.471	0.196	0.488	0.304	0.241
MCA, mm^2^	250.6 (97.5)	232.1 (104.7)	249.6 (113.4)	240.1 (135.7)	0.827	0.238	0.504	0.217	0.356	0.462	0.539
PNS-AP	27.8 (4.2)	26.5 (3.8)	27.7 (4.4)	26.5 (4.0)	0.639	0.455	0.243	0.767	0.589	0.610	0.371
PNS-lateral	37.3 (5.7)	38.3 (6.3)	39.0 (5.2)	37.8 (5.1)	0.405	0.284	0.807	0.148	0.186	0.918	0.203
PNS-AP/ lateral	0.71 (0.12)	0.68 (0.13)	0.69 (0.14)	0.72 (0.17)	0.640	0.286	0.480	0.884	0.708	0.312	0.477
E-AP	13.4(2.7)	12.3 (3.9)	12.8 (3.7)	12.5 (3.1)	0.640	0.274	0.433	0.252	0.706	0.865	0.796
E- lateral	30.3(3.0)	30.5 (3.4)	31.4 (2.8)	31.1 (3.7)	0.421	0.994	0.160	0.284	0.217	0.325	0.935
E-AP/lateral	0.43 (0.11)	0.40 (0.12)	0.40 (0.11)	0.40 (0.11)	0.584	0.301	0.244	0.234	0.846	0.875	0.963
**Hyoid**											
H-Eb	8.7(1.5)	8.6(3.1)	7.9(1.9)	7.5(1.7)	0.125	0.264	0.035	0.029	0.631	0.395	0.813
H-Me	44.4(4.7)	45.1(4.9)	44.1(5.1)	42.3(3.7)	0.109	0.541	0.859	0.060	0.448	0.013	0.110
H-C3	28.0(3.0)	26.5(3.0)	27.0(3.0)	26.0(3.4)	0.083	0.054	0.205	0.019	0.485	0.591	0.230
H-X	9.8(6.3)	7.2(7.2)	6.9(5.4)	3.9(5.3)	0.004*	0.146	0.065	0.000*	0.862	0.050	0.036
H-Y	93.2(5.8)	95.5(5.3)	95.0(4.7)	93.2(7.0)	0.489	0.160	0.252	0.935	0.941	0.336	0.420

^*^P < 0.05

^a^Results of One-way analysis of variance

Table 4 compares oropharynx and hyoid bone changes from T0 to T1 among the four skeletal pattern subgroups. Oropharynx volumes increased significantly in the class I-norm and class I-hyper subgroups. Although there was a tendency for increased oropharynx volumes in class II-norm and class II-hyper subgroups, these changes were not statistically significant. Furthermore, the increase of oropharynx volume in class I-norm subgroup was more significant than the increase of oropharynx volume in class II-norm subgroup, indicating that the sagittal skeletal pattern could affect the influence of extraction on oropharynx volume. The increase of MCA in all subgroups were not significant, but it differed significantly between class I-hyper and class II-hyper subgroups. With regard to oropharynx morphology, the PNS- lateral increased significantly in class I-norm, class I-hyper, and class II-norm patients; the PNS-AP increased significantly in class I-hyper and class II-hyper patients. No significant differences among subgroups were observed in any hyoid bone variables from T0 to T1, while the change in H-Y distance significantly differed between class I-hyper patients and class II-hyper patients.

**Table 4:** Comparison of the changes in oropharynx and hyoid bone position among the extraction patients with different skeletal patterns

Variable	Class I-norm			Class I-hyper			Class II-norm			Class II-hyper			Class I-norm vs Class I-hyper	Class I-norm vs Class II-norm	Class I-hyper vs Class II-hyper	Class II-norm vs Class II-hyper
	T0 Mean (SD)	T1 Mean (SD)	***p***	T0 Mean (SD)	T1 Mean (SD)	***p***	T0 Mean (SD)	T1 Mean (SD)	***p***	T0 Mean (SD)	T1 Mean (SD)	***p***	***p***	***p***	***p***	***p***
**Oropharynx**																
Vol, mm^3^	16,393.0 (5127.0)	18,756.0 (7254.5)	0.035*	16,270.2 (5429.6)	18,552.4 (6312.2)	0.007*	16,708.3 (6318.0)	17,405.3 (5824.7)	0.345	15,123.3 (5238.0)	16,414.8 (6020.5)	0.089	0.511	0.029*	0.623	0.866
MCA, mm^2^	250.6 (97.5)	288.1 (158.0)	0.176	232.1 (104.7)	264.8 (109.4)	0.084	249.6 (113.4)	263.7 (107.1)	0.405	241.0 (135.7)	250.4 (121.4)	0.687	0.764	0.085	0.033*	0.231
PNS-AP	27.8 (4.2)	28.5 (2.9)	0.265	26.5 (3.8)	27.7 (3.3)	0.018*	27.7 (4.4)	28.1 (3.3)	0.446	26.5 (4.0)	27.6 (4.3)	0.050*	0.457	0.130	0.086	0.184
PNS-lateral	37.3 (5.7)	40.0 (7.2)	0.006*	38.3 (6.3)	40.0 (6.1)	0.001*	39.0 (5.2)	40.1 (4.6)	0.016*	37.8 (5.1)	38.7 (5.6)	0.129	0.835	0.238	0.615	0.136
PNS-AP/ lateral	0.71 (0.12)	0.73 (0.11)	0.237	0.68 (0.13)	0.70 (0.12)	0.797	0.69 (0.14)	0.68 (0.07)	0.299	0.72 (0.17)	0.72 (0.10)	0.441	0.348	0.690	0.469	0.160
E-AP	13.4 (2.7)	13.5 (3.8)	0.862	12.3 (3.9)	12.5 (4.1)	0.843	12.8 (3.7)	13.0 (3.6)	0.716	12.5 (3.1)	13.0 (3.5)	0.326	0.06	0.158	0.591	0.287
E- lateral	30.3 (3.0)	31.4 (3.5)	0.034*	30.5 (3.4)	31.2 (3.3)	0.148	31.4 (2.8)	31.7 (3.0)	0.481	31.1 (3.7)	31.6 (3.4)	0.153	0.312	0.456	0.738	0.781
E-AP/lateral	0.44 (0.09)	0.43 (0.10)	0.171	0.40 (0.12)	0.42 (0.10)	0.465	0.40 (0.10)	0.41 (0.10)	0.719	0.40 (0.09)	0.41 (0.11)	0.349	0.176	0.425	0.976	0.584
**Hyoid**																
H-Eb	8.7(1.5)	8.5 (1.9)	0.750	8.6(3.1)	9.0 (2.8)	0.147	7.9(1.9)	7.7 (1.5)	0.470	7.5(1.7)	7.6 (1.7)	0.678	0.186	0.988	0.333	0.450
H-Me	44.4(4.7)	46.0 (5.6)	0.181	45.1(4.9)	47.0 (6.6)	0.051	44.1(5.1)	44.5 (5.9)	0.580	42.3(3.7)	43.0 (4.2)	0.229	0.300	0.315	0.355	0.397
H-C3	28.0(3.0)	28.0 (3.2)	0.965	26.5(3.0)	26.9 (3.1)	0.413	27.0(3.0)	27.0 (3.1)	0.918	26.0(3.4)	26.7 (2.6)	0.117	0.556	0.922	0.655	0.190
H-X	9.8(6.3)	8.2 (6.4)	0.142	7.2(7.2)	5.5 (7.4)	0.060	6.9(5.4)	6.5 (6.9)	0.637	3.9(5.3)	3.7 (7.0)	0.720	0.950	0.361	0.236	0.934
H-Y	93.2(5.8)	93.3 (5.2)	0.919	95.5(5.3)	96.5 (5.0)	0.103	95.0(4.7)	95.5 (5.4)	0.372	93.2(7.0)	92.7 (6.6)	0.221	0.492	0.706	0.041*	0.149

^*^P < 0.05

### Comparisons of oropharynx and hyoid bone changes between maximum and moderate retraction subgroups

Extraction patients were divided into two subgroups based on anterior teeth retraction status: 48 maximum retraction patients (ΔU1-X ≥ 6 mm) and 72 moderate retraction patients (3 mm < ΔU1-X < 6 mm). The mean changes in U1-X were 6.8 mm in the maximum retraction group and 4.1 mm in the moderate retraction group. As shown in Table 5, oropharynx volume and MCA from T0 to T1 increased significantly in the moderate retraction group, but not in the maximum retraction group. The PNS-AP, PNS-lateral, and E-lateral from T0 to T1 increased significantly in both groups. None of the changes in oropharynx variables differed significantly between the two groups. With regard to hyoid bone, the H-Me and H-C3 distance increased significantly in the maximum retraction group, while the H-X decreased significantly. In the moderate retraction group, no significant changes were observed in the hyoid bone position from T0 to T1. Overall, these changes indicate greater posterior movement of the hyoid bone in the maximum retraction group.

**Table 5:** Comparison of oropharynx and hyoid bone position between the maximum and moderate retraction groups

Variable	Maximum retraction group (***n*** = 48)			Moderate retraction group (***n*** = 72)			Maximum retraction group vs Moderate retraction group
	T0 Mean (SD)	T1 Mean (SD)	***P***	T0 Mean (SD)	T1 Mean (SD)	***P***	***P***
**Oropharynx**							
Vol, mm^3^	15,929.9 (4897.7)	17,135.1 (6426.2)	0.062	16,252.9 (5913.9)	17,780.1 (6369.7)	0.003*	0.219
MCA, mm^2^	243.4 (121.8)	263.0 (143.1)	0.353	243.3 (106.8)	269.3 (112.0)	0.045*	0.083
PNS-AP	26.7 (3.9)	27.7 (3.4)	0.022*	27.4 (4.3)	28.1 (3.5)	0.033*	0.739
PNS-lateral	38.7 (3.9)	39.9 (4.6)	0.002*	37.7 (6.4)	39.4 (6.7)	0.000*	0.128
PNS-AP/ lateral	0.69 (0.09)	0.70 (0.07)	0.572	0.74 (0.11)	0.73 (0.12)	0.167	0.154
E-AP	12.2 (3.3)	12.7 (4.3)	0.290	13.1 (3.3)	13.2 (3.4)	0.869	0.458
E- lateral	31.2 (3.5)	31.9 (3.7)	0.044*	30.5 (3.0)	31.2 (2.9)	0.030*	0.691
E-AP/lateral	0.39 (0.11)	0.40 (0.12)	0.729	0.43 (0.10)	0.42 (0.10)	0.465	0.962
**Hyoid**							
H-Eb	8.3 (2.0)	8.4 (2.3)	0.660	8.4 (2.5)	8.2 (1.9)	0.852	0.441
H-Me	43.3 (4.2)	45.5 (6.3)	0.004*	43.9 (5.3)	45.0 (5.2)	0.078	0.664
H-C3	26.5 (3.3)	27.5 (3.4)	0.003*	27.3 (3.0)	27.1 (2.9)	0.507	0.0.95
H-X	8.5 (5.8)	6.7 (6.9)	0.008*	6.1 (6.5)	5.1 (7.0)	0.137	0.880
H-Y	93.2 (5.8)	94.0 (5.8)	0.061	94.8 (5.6)	94.7 (5.3)	0.814	0.044*

^*^P < 0.05

## Discussion

This study compared oropharynx and hyoid bone changes between female extraction patients and female non-extraction patients; it also explored differences in oropharynx and hyoid bone changes among four skeletal patterns. Female extraction patients exhibited increases in oropharynx volume and posterior movement of the hyoid bone compared with female non-extraction patients; the increase in oropharynx volume was greater in skeletal class I patients and moderate retraction patients.

Extraction treatment can alleviate crowding and reduce facial convexity. However, an important concern during dental extraction is respiratory function, particularly in the upper airway (i.e. nasopharynx, oropharynx, hypopharynx). The nasopharynx and hypopharynx are supported by bone and cartilage, and are located far from the oral cavity; they are not easily influenced by extraction treatment. In contrast, the oropharynx comprises soft tissue and tongue; it is directly connected to the oral cavity. Besides, Jason et al. assessed oropharyngeal airway volume and demonstrated excellent intra-examiner and inter-examiner reliabilities, while the reliabilities of nasopharynx and hypopharynx assessments via CBCT have been low [18]. Hence, the oropharynx was the main focus of measurements in the present study.

Several previous studies have used CBCT to analyse the effect of extraction on upper airway size. However, their results have been inconsistent [5, 9, 10, 17, 19]. Sun et al. and Zheng et al. both reported significant decreases in oropharynx volume after maximum incisor retraction in class I bimaxillary protrusion patients [20, 21]. They presumed that dental extraction reduced arch length and oral cavity, which led to posterior movement of the tongue, followed by oropharyngeal narrowing. In contrast, Joy et al. and Pliska et al. [17, 19]. reported no clinically significant changes in oropharynx volume. Heterogeneity in patient characteristics (e.g. age, sex, skeletal pattern, extraction indication) among the studies might have contributed to these disparate findings. At present, no strong evidence is available regarding the effects of extraction on upper airway size. In this retrospective study with a large sample size, we found that oropharyngeal changes did not differ significantly between extraction and non-extraction groups, consistent with reports by Joy et al. and Pliska et al. [17, 19]. However, we also found that the oropharynx volume in the extraction group significantly increased from T0 to T1. Notably, the transverse diameter of the oropharynx (PNS-lateral and E-lateral) exhibited greater changes in the extraction group, indicating that the lateral wall of the oropharynx is easily changed. Da Costa et al. suggested that the lateral wall of oropharynx was the main area affected during orthognathic surgery and orthodontic treatment [22].

An enlarged upper airway after extraction has been reported previously in adolescent patients because of ongoing craniofacial growth; this change has rarely been reported in adults. Several factors might contribute to an increased oropharynx size in female adults. First, class II elastics were commonly used in the extraction group to reinforce the maxillary anchorage. Class II elastics could move the mandible forward, while increasing the oropharynx size. Second, patients with bimaxillary protrusion usually present with a narrow dental arch [23]. The constricted dental arch could lead to tongue crowding, which might be associated with a narrow oropharynx. After extraction treatment, the expanded dental arch leads to a larger oropharynx. Increased upper airway dimensions have been reported after maxillary expansion [24, 25]. Finally, the mesial movement of posterior teeth, which provides a posterior space for the tongue, could lead to greater airway volume. In the present study, we found that increases in the oropharynx volume and MCA were greater in the moderate retraction group than in the maximum retraction group, which confirmed this hypothesis. The mechanism of oropharyngeal changes should be investigated in future studies.

Some studies have theorized that dental extraction can reduce upper airway size and increases the risk of obstructive sleep apnoea (OSA), but there are inherent disadvantages as these were performed with lateral cephalogram [6–8, 14, 15]. The sagittal information provided by lateral cephalogram may be misleading. Furthermore, the upper airway varies among patients, and previous studies have included small sample sizes. In contrast, we included a larger sample size and found larger oropharynx size upon CBCT analysis. It is also important to note that there is no direct link between a narrow airway and OSA. The American Association of Orthodontists has indicated that a narrow airway does not result in OSA [26]. CBCT cannot provide information regarding airway function; OSA should be diagnosed via polysomnography or an out-of-centre sleeping test. Extraction safety in OSA patients should be investigated in future studies.

The hyoid bone is connected with the pharynx, cranial base, and mandibular symphysis via muscles and ligaments; this complex can maintain airway stability. In the present study, we found significant posterior movement of the hyoid bone in the extraction group and slight anterior movement of the hyoid bone in the non-extraction group, especially in class I-hyper patients; posterior movement of the hyoid bone was greater in the maximum retraction group than in the moderate retraction group. The vertical position of the hyoid bone was stable in all groups. Our results are consistent with those of Bhatia et al. [6], who reported that the hyoid bone moved posteriorly during anterior teeth retraction. In contrast, Maaitah et al. and Germec-Cakan et al. [7, 8]. reported no significant changes in hyoid bone position. Although many studies have reported associations between the hyoid bone and oropharynx, the mechanism of hyoid bone compensation during oropharyngeal changes remains unclear. Wang et al. suggested that the hyoid bone moves both posteriorly and inferiorly after extraction treatment, which could prevent encroachment of the tongue into the oropharynx [27].

The differences in oropharynx among patients with distinct skeletal patterns have been reported previously [11–13]. With regard to the vertical skeletal patterns, the hyperdivergent patients had smaller upper airway volumes compared with normodivergent and hypodivergent patients [11]. With regard to sagittal skeletal patterns, the skeletal class II patients had a narrow upper airway [13, 28]. Our results confirmed that skeletal class II and hyperdivergent patients had a narrow oropharynx volume compared with other skeletal pattern patients. To our knowledge, dental extraction involving the oropharynx has not been investigated among patients with distinct skeletal patterns. We observed significant differences in premolar extraction effects on oropharynx volume (between class I-norm and class II-norm patients) and MCA of the oropharynx (between class I-hyper and class II-hyper patients). The oropharynx volumes from T0 to T1 increased significantly in the class I group, but not in the class II group. These results suggest that, compared with vertical skeletal patterns, the effects of sagittal skeletal patterns might be more important in the context of oropharyngeal changes in female patients.

Class II and hyperdivergent patients reportedly have a narrow upper airway and a high risk of OSA [29, 30]. In our study, class II and hyperdivergent patients with a narrow oropharynx and posterior hyoid bone position did not exhibit oropharynx collapse, but they tended to exhibit increases in oropharynx volume and MCA after extraction treatment. Zhang et al. also analysed the effects of extraction on upper airway changes in skeletal class II and hyperdivergent patients; consistent with our results, they found that the volume and MCA did not change significantly [10]. Hence, changes in oropharynx variables might not be associated with baseline condition; skeletal class II and hyperdivergent patients without OSA did not exhibit contraindications for premolar extraction.

In addition to skeletal pattern, changes in the upper airway after extraction treatment may have been inconsistent among previous studies because of variations in anterior teeth retraction. The extraction space could be closed by anterior teeth retraction and posterior teeth mesial movement. We divided extraction patients into maximum and moderate retraction groups based on changes in U1-X. Changes in oropharynx variables did not differ significantly between groups, consistent with a study by Valiathan et al., who found that changes in incisor angulation and position did not cause significant differences in oropharynx volumes [31]. In the present study, the volume and MCA of the oropharynx from T0 to T1 increased significantly in the moderate retraction group, but not in the maximum retraction group. This finding was consistent with our expectations because the maxillary mini-screw was commonly used in the maximum retraction group to achieve considerable retraction of anterior teeth, while class II elastic was commonly used in the moderate retraction group. The mesial movement of posterior teeth and the use of class II elastic might have contributed to a significant increase in oropharynx size in the moderate retraction group.

There are some limitations in this study. Firstly, the number of non-extraction patients was limited in this study due to ethical considerations. We used CBCT to confirm the amount of anterior tooth retraction in extraction patients according to pre-treatment alveolar bone morphology; teeth were kept in alveolar bone after orthodontic treatment. Most non-extraction patients in our study used CBCT only because of factors related to implant restoration and impacted teeth, which caused a small sample size of control group. Secondly, the airway could be easily influenced by factors such as age, sex, weight, head position, breathing mode, and tongue position [32, 33]. The upper airway size increases until 20 years of age and decreases thereafter [34, 35]. Our study population was limited to fully grown females in an attempt to avoid these confounding factors. While, the OSA is more prevalent in male patients than in female patients [36]. In this study, we only included female patients. The effects of first premolar extraction on the oropharynx and hyoid bone positions in male patients should be further investigated. Besides, body mass index was not recorded after orthodontic treatment, so the effects of weight changes during orthodontic treatment could not be analysed. Thirdly, due to the relatively low prevalence of four premolar extraction in skeletal class III patients and hypodivergent patients in Asian, only skeletal class I and class II patients with hyperdivergent and normodivergent patterns were included. Importantly, the ability for upper airway adaptation should be considered when interpreting our findings. Our results only reflect changes in the size and morphology of the oropharynx and the hyoid bone position in female extraction patients. Respiratory function and the long-term stabilities of the oropharynx and hyoid bone should be investigated in future studies.

## Conclusion

- In female adult patients without snoring and OSA, first premolar extraction tended to increase the oropharynx volume and caused posterior movement of the hyoid bone.
- Oropharynx volume increased significantly after orthodontic extraction treatment in skeletal class I patients, but not in skeletal class II patients.
- In skeletal class II and hyperdivergent patients with a narrow oropharynx, first premolar extraction did not negatively influence oropharynx size or hyoid bone position.
- No significant differences were observed in oropharyngeal changes between moderate retraction patients and maximum retraction patients.

## Supplementary Information

**Additional file 1: Supplementary Table 1.** Comparison of the changes in oropharynx and hyoid bone position between class I-norm extraction patients and class I-norm non-extraction patients.

**Additional file 2: Supplementary Table 2.** Comparison of the changes in oropharynx and hyoid bone position between class II-norm extraction patients and class II-norm non-extraction patients.

**Additional file 3: Supplementary Table 3.** Comparison of the changes in oropharynx and hyoid bone position between class I-hyper extraction patients and class I- hyper non-extraction patients.

**Additional file 4: Supplementary Table 4.** Comparison of the changes in oropharynx and hyoid bone position between class II-hyper extraction patients and class II- hyper non-extraction patients.

## Data Availability

All data analyzed during the current study are included in this published article. Any other request about the data, contact e-mail: weiranli@bjmu.edu.cn.
