# Supplementary material for: Oropharynx and hyoid bone changes in female extraction patients with distinct sagittal and vertical skeletal patterns: a retrospective study
Source: Head Face Med. 2022 Sep 5;18:31. doi: 10.1186/s13005-022-00334-1 (PMC9442905; doi:10.1186/s13005-022-00334-1)
Supplement: Supplementary file 1 — Additional file 1: Supplementary Table 1. Comparison of the changes in oropharynx and hyoid bone position between class I-norm extraction patients and class I-norm non-extraction patients. [file 13005_2022_334_MOESM1_ESM.docx]

Supplementary Table 1. Comparison of the changes in oropharynx and hyoid bone position between class I-norm extraction patients and class I-norm non-extraction patients

| **Variable** | **Class I-norm**  **extraction patients**  **(n=30)** | | | **Class I-norm**  **non-extraction patients**  **(n=10)** | | | ***p*** |
| --- | --- | --- | --- | --- | --- | --- | --- |
|  | **T0**  **Mean (SD)** | **T1**  **Mean (SD)** | ***p*** | **T0**  **Mean (SD)** | **T1**  **Mean (SD)** | ***p*** |  |
| **Oropharynx** |  |  |  |  |  |  |  |
| Vol, mm^3^ | 16393.0 (5127.0) | 18756.0 (7254.5) | 0.035* | 14452.1 (3322.6) | 15733.1 (5261.4) | 0.445 | 0.571 |
| MCA, mm^2^ | 250.6 (97.5) | 288.1 (158.0) | 0.176 | 208.1 (65.0) | 237.9 (139.1) | 0.364 | 0.881 |
| PNS-AP | 27.8 (4.2) | 28.5 (2.9) | 0.265 | 26.5 (4.1) | 27.8 (3.5) | 0.139 | 0.547 |
| PNS-lateral | 37.3 (5.7) | 40.0 (7.2) | 0.006** | 35.8 (6.3) | 36.9 (6.4) | 0.464 | 0.818 |
| PNS-AP/ lateral | 0.71 (0.12) | 0.73 (0.11) | 0.237 | 0.75 (0.09) | 0.76 (0.10) | 0.721 | 0.213 |
| E-AP | 13.4 (2.7) | 13.5 (3.8) | 0.862 | 11.8 (2.8) | 13.0 (4.6) | 0.407 | 0.465 |
| E- lateral | 30.3 (3.0) | 31.4 (3.5) | 0.034* | 28.7 (3.1) | 28.7 (5.1) | 0.916 | 0.238 |
| E-AP/lateral | 0.44 (0.09) | 0.43 (0.10) | 0.171 | 0.41 (0.09) | 0.45 (0.14) | 0.289 | 0.114 |
| **Hyoid** |  |  |  |  |  |  |  |
| H-Eb | 8.7（1.5） | 8.5 (1.9) | 0.750 | 9.6 (3.7) | 8.1 (3.0) | 0.130 | 0.194 |
| H-Me | 44.4（4.7） | 46.0 (5.6) | 0.181 | 46.9 (4.1) | 45.7 (4.6) | 0.472 | 0.107 |
| H-C3 | 28.0（3.0） | 28.0 (3.2) | 0.965 | 28.8 (3.6) | 27.4 (5.1) | 0.386 | 0.396 |
| H-X | 9.8（6.3） | 8.2 (6.4) | 0.142 | 5.4 (4.1) | 6.8 (3.7) | 0.321 | 0.087 |
| H-Y | 93.2（5.8） | 93.3 (5.2) | 0.919 | 97.2 (6.3) | 96.2 (6.9) | 0.308 | 0.479 |

**P*<0.05
