# Supplementary material for: Oropharynx and hyoid bone changes in female extraction patients with distinct sagittal and vertical skeletal patterns: a retrospective study
Source: Head Face Med. 2022 Sep 5;18:31. doi: 10.1186/s13005-022-00334-1 (PMC9442905; doi:10.1186/s13005-022-00334-1)
Supplement: Supplementary file 2 — Additional file 2: Supplementary Table 2. Comparison of the changes in oropharynx and hyoid bone position between class II-norm extraction patients and class II-norm non-extraction patients. [file 13005_2022_334_MOESM2_ESM.docx]

Supplementary Table 2. Comparison of the changes in oropharynx and hyoid bone position between class II-norm extraction patients and class II-norm non-extraction patients

| **Variable** | **Class II-norm**  **extraction patients**  **(n=30)** | | | **Class II-norm**  **non-extraction patients**  **(n=10)** | | | ***p*** |
| --- | --- | --- | --- | --- | --- | --- | --- |
|  | **T0**  **Mean (SD)** | **T1**  **Mean (SD)** | ***p*** | **T0**  **Mean (SD)** | **T1**  **Mean (SD)** | ***p*** |  |
| **Oropharynx** |  |  |  |  |  |  |  |
| Vol, mm^3^ | 16708.3 (6318.0) | 17405.3 (5824.7) | 0.345 | 16140.3 (5629.3) | 14552.4 (3807.0) | 0.402 | 0.167 |
| MCA, mm^2^ | 249.6 (113.4) | 263.7 (107.1) | 0.405 | 234.8 (112.2) | 200.4 (67.7) | 0.358 | 0.158 |
| PNS-AP | 27.7 (4.4) | 28.1 (3.3) | 0.016* | 29.2 (2.9) | 29.4 (3.6) | 0.564 | 0.770 |
| PNS-lateral | 39.0 (5.2) | 40.1 (4.6) | 0.446 | 39.5 (4.1) | 40.8 (4.5) | 0.589 | 0.940 |
| PNS-AP/ lateral | 0.69 (0.14) | 0.68 (0.07) | 0.299 | 0.74 (0.08) | 0.71 (0.09) | 0.196 | 0.346 |
| E-AP | 12.8 (3.7) | 13.0 (3.6) | 0.716 | 12.4 (4.0) | 11.7 (3.1) | 0.410 | 0.378 |
| E- lateral | 31.4 (2.8) | 31.7 (3.0) | 0.481 | 32.1 (3.7) | 31.0 (2.9) | 0.220 | 0.067 |
| E-AP/lateral | 0.40 (0.10) | 0.41 (0.10) | 0.719 | 0.38 (0.11) | 0.38 (0.10) | 0.823 | 0.162 |
| **Hyoid** |  |  |  |  |  |  |  |
| H-Eb | 7.9（1.9） | 7.7 (1.5) | 0.470 | 8.6 (1.3) | 8.1 (1.1) | 0.146 | 0.363 |
| H-Me | 44.1（5.1） | 44.5 (5.9) | 0.580 | 45.5 (4.3) | 44.9 (7.3) | 0.711 | 0.116 |
| H-C3 | 27.0（3.0） | 27.0 (3.1) | 0.918 | 26.7 (2.8) | 27.3 (3.6) | 0.541 | 0.513 |
| H-X | 6.9（5.4） | 6.5 (6.9) | 0.637 | 9.3 (7.0) | 10.6 (8.1) | 0.455 | 0.219 |
| H-Y | 95.0（4.7） | 95.5 (5.4) | 0.372 | 94.3 (6.8) | 95.8 (7.5) | 0.041* | 0.231 |

**P*<0.05
