# Supplementary material for: Oropharynx and hyoid bone changes in female extraction patients with distinct sagittal and vertical skeletal patterns: a retrospective study
Source: Head Face Med. 2022 Sep 5;18:31. doi: 10.1186/s13005-022-00334-1 (PMC9442905; doi:10.1186/s13005-022-00334-1)
Supplement: Supplementary file 3 — Additional file 3: Supplementary Table 3. Comparison of the changes in oropharynx and hyoid bone position between class I-hyper extraction patients and class I- hyper non-extraction patients. [file 13005_2022_334_MOESM3_ESM.docx]

Supplementary Table 3. Comparison of the changes in oropharynx and hyoid bone position between class I-hyper extraction patients and class I- hyper non-extraction patients

| **Variable** | **Class I-hyper**  **extraction patients**  **(n=30)** | | | **Class I-hyper**  **non-extraction patients**  **(n=10)** | | | ***p*** |
| --- | --- | --- | --- | --- | --- | --- | --- |
|  | **T0**  **Mean (SD)** | **T1**  **Mean (SD)** | ***p*** | **T0**  **Mean (SD)** | **T1**  **Mean (SD)** | ***p*** |  |
| **Oropharynx** |  |  |  |  |  |  |  |
| Vol, mm^3^ | 16270.2 (5429.6) | 18552.4 (6312.2) | 0.007** | 18784.5 (7471.2) | 19514.0 (6998.2) | 0.626 | 0.338 |
| MCA, mm^2^ | 232.1 (104.7) | 264.8 (109.4) | 0.084 | 262.8 (130.7) | 262.5 (96.5) | 0.994 | 0.366 |
| PNS-AP | 26.5 (3.8) | 27.7 (3.3) | 0.018* | 28.4 (5.2) | 29.5 (4.1) | 0.236 | 0.913 |
| PNS-lateral | 38.3 (6.3) | 40.0 (6.1) | 0.001** | 39.9 (5.3) | 41.5 (4.5) | 0.188 | 0.959 |
| PNS-AP/ lateral | 0.68 (0.13) | 0.70 (0.12) | 0.797 | 0.71 (0.06) | 0.71 (0.08) | 0.761 | 0.896 |
| E-AP | 12.3 (3.9) | 12.5 (4.1) | 0.843 | 12.6 (4.4) | 13.5 (4.0) | 0.417 | 0.928 |
| E- lateral | 30.5 (3.4) | 31.2 (3.3) | 0.148 | 32.1 (3.6) | 32.8 (3.8) | 0.416 | 0.991 |
| E-AP/lateral | 0.40 (0.12) | 0.42 (0.10) | 0.465 | 0.39 (0.11) | 0.40 (0.08) | 0.455 | 0.882 |
| **Hyoid** |  |  |  |  |  |  |  |
| H-Eb | 8.6（3.1） | 9.0 (2.8) | 0.147 | 8.3 (1.4) | 8.2 (1.2) | 0.655 | 0.246 |
| H-Me | 45.1（4.9） | 47.0 (6.6) | 0.051 | 46.7 (5.0) | 45.3 (3.7) | 0.403 | 0.100 |
| H-C3 | 26.5（3.0） | 26.9 (3.1) | 0.413 | 27.5 (3.5) | 26.8 (3.1) | 0.518 | 0.239 |
| H-X | 7.2（7.2） | 5.5 (7.4) | 0.060 | 6.3 (7.1) | 9.3 (4.8) | 0.267 | 0.050* |
| H-Y | 95.5（5.3） | 96.5 (5.0) | 0.103 | 97.2 (7.4) | 90.0 (22.8) | 0.508 | 0.167 |

**P*<0.05
