# Supplementary material for: Oropharynx and hyoid bone changes in female extraction patients with distinct sagittal and vertical skeletal patterns: a retrospective study
Source: Head Face Med. 2022 Sep 5;18:31. doi: 10.1186/s13005-022-00334-1 (PMC9442905; doi:10.1186/s13005-022-00334-1)
Supplement: Supplementary file 4 — Additional file 4: Supplementary Table 4. Comparison of the changes in oropharynx and hyoid bone position between class II-hyper extraction patients and class II- hyper non-extraction patients. [file 13005_2022_334_MOESM4_ESM.docx]

Supplementary Table 4. Comparison of the changes in oropharynx and hyoid bone position between class II-hyper extraction patients and class II- hyper non-extraction patients

| **Variable** | **Class II-hyper**  **extraction patients**  **(n=30)** | | | **Class II-hyper**  **non-extraction patients**  **(n=10)** | | | ***p*** |
| --- | --- | --- | --- | --- | --- | --- | --- |
|  | **T0**  **Mean (SD)** | **T1**  **Mean (SD)** | ***p*** | **T0**  **Mean (SD)** | **T1**  **Mean (SD)** | ***p*** |  |
| **Oropharynx** |  |  |  |  |  |  |  |
| Vol, mm^3^ | 15123.3 (5238.0) | 16414.8 (6020.5) | 0.089 | 16303.9 (5288.9) | 16685.3 (5575.3) | 0.641 | 0.506 |
| MCA, mm^2^ | 241.0 (135.7) | 250.4 (121.4) | 0.687 | 240.0 (110.0) | 234.6 (84.6) | 0.851 | 0.612 |
| PNS-AP | 26.5 (4.0) | 27.6 (4.3) | 0.050* | 28.8 (4.2) | 28.9 (3.7) | 0.678 | 0.351 |
| PNS-lateral | 37.8 (5.1) | 38.7 (5.6) | 0.129 | 40.3 (7.2) | 40.9 (6.7) | 0.453 | 0.331 |
| PNS-AP/ lateral | 0.72 (0.17) | 0.72 (0.10) | 0.441 | 0.72 (0.06) | 0.71 (0.07) | 0.959 | 0.532 |
| E-AP | 12.5 (3.1) | 13.0 (3.5) | 0.326 | 12.9 (3.6) | 12.5 (3.1) | 0.612 | 0.379 |
| E- lateral | 31.1 (3.7) | 31.6 (3.4) | 0.153 | 31.5 (2.9) | 31.4 (2.8) | 0.886 | 0.383 |
| E-AP/lateral | 0.40 (0.09) | 0.41 (0.11) | 0.349 | 0.41 (0.12) | 0.40 (0.10) | 0.646 | 0.458 |
| **Hyoid** |  |  |  |  |  |  |  |
| H-Eb | 7.5（1.7） | 7.6 (1.7) | 0.678 | 9.8 (3.3) | 10.5 (3.6) | 0.332 | 0.214 |
| H-Me | 42.3（3.7） | 43.0 (4.2) | 0.229 | 44.5 (6.0) | 44.9 (4.2) | 0.853 | 0.591 |
| H-C3 | 26.0（3.4） | 26.7 (2.6) | 0.117 | 28.6 (3.5) | 27.9 (3.3) | 0.318 | 0.187 |
| H-X | 3.9（5.3） | 3.7 (7.0) | 0.720 | 10.3 (9.7) | 9.0 (10.5) | 0.525 | 0.791 |
| H-Y | 93.2（7.0） | 92.7 (6.6) | 0.221 | 94.1 (6.6) | 93.1 (6.6) | 0.304 | 0.500 |

**P*<0.05
